# Efficacy and Safety of Blood Transfusion Protocols in the Treatment of Myocardial Infarction: A Review of Restrictive and Liberal Approaches

**DOI:** 10.7759/cureus.78307

**Published:** 2025-01-31

**Authors:** Simhachalam Gurugubelli, Ravi Venkata Sai Krishna Medarametla, Ujwala Koduru, Arvind Kunadi

**Affiliations:** 1 Internal Medicine, Memorial Healthcare, Gulfport, USA; 2 Internal Medicine, Mamata Medical College, Khammam, IND; 3 Internal Medicine, McLaren Greater Lansing, Lansing, USA; 4 Internal Medicine/Nephrology, McLaren Health Care/Michigan State University (MSU), Flint, USA

**Keywords:** acute myocardial infarction (ami), anemia in myocardial infarction, blood transfusion, blood transfusion in myocardial infarction, evidence-based transfusion guidelines, hemoglobin levels, liberal transfusion strategy, restrictive transfusion strategy, safety of transfusion, transfusion protocols

## Abstract

Acute myocardial infarction (AMI) is a leading cause of death worldwide, and anemia in patients following AMI is quite common. Blood transfusions are one means of treating anemia, but once again, it is surrounded by debate over the best approach for transfusion: whether it is restrictive or liberal. This review assesses the efficacy and safety of a restrictive versus liberal blood transfusion strategy in AMI patients. Literature searches of the existing database were made with a view to retrieving RCTs, meta-analyses, systematic reviews, and clinical practice guidelines pertaining to both restrictive and liberal transfusion strategies for comparison of outcomes. Evidence suggests that this restrictive approach brings no patient harm, except perhaps in high-risk subgroups such as larger cardiovascular comorbidities. Key trials have shown that a restrictive strategy is at least as effective as a liberal strategy for most patients, including TRICC, TRACS, FOCUS, MINT, TITRe2, and REALITY Trials. Specifically, particular populations, especially those with pre-existing heart disease, may benefit from higher hemoglobin thresholds to prevent adverse outcomes. The best transfusion strategy should be tailored for each patient based on his/her personal factors, above all in cardiovascular health. A more restrictive transfusion strategy was effective and safe for the general population, but the subgroup of patients with very poor cardiovascular disease may require a more liberal approach. Further studies with better management guidelines are warranted to guide transfusion practices for optimal care in AMI patients.

## Introduction and background

Acute myocardial infarction (AMI) is one of the important causes of death in developed societies. Its prevalence is nearly three million people worldwide, while over one million deaths are reported annually in the United States [1-3]. Myocardial infarction (MI) causes irreversible necrosis of the myocardium due to a lack of adequate oxygen supply. Patients with MI face a risk of diastolic and systolic dysfunction, which creates an opportunity for the incidence of arrhythmia. Additionally, MI can lead to severe complications. Reperfusion or resuming blood flow to the heart should be performed as early as possible.

Anemia following AMI usually results from a reduction in red blood cells, which follows invasive procedures and the use of anticoagulants [4] and causes inadequate delivery of oxygen to tissues. Anemia is reported in 15 to 43% of patients with MI [5]. This drastically changes the patient's health status and increases the possible death in both the short term and long term after a heart attack event [5,6]. In most cases, this will prompt a clinician to offer a blood transfusion to reverse the oxygen deficit in patients with an AMI with anemia since this quickly restores the red cell count and reverses the symptoms [7]. However, the amount of blood transfusion is more critical, as too much transfusion brings along a set of side effects, such as cardiac or pulmonary overload, besides increasing the risk for the formation of blood clots, which further may deteriorate the state of the patient [8-10].

Transfusion strategies about this background are becoming very controversial among the medical community [11-13]. Its supporters insist on early and more frequent initiation of transfusion, while those of the "restrictive" approach take a conservative path in managing this population. A restrictive transfusion strategy aims to maintain a lower hemoglobin, usually between 70 g/L and 90 g/L; the transfusion trigger would be when hemoglobin drops below 70 g/L. On the other hand, the liberal transfusion strategy is aimed at maintaining higher hemoglobin, usually between 100 g/L and 120 g/L, in which the threshold for transfusion is when hemoglobin falls below 100 g/L [14].

Although prior attempts at research have been conducted without offering resounding guidance, the findings in some of the more extensive studies pointed toward the non-inferiority of the restrictive approach up to 30 days post-AMI [15]. A significant gap remains on which strategy appropriately serves AMI patients with anemia.

## Review

Blood transfusion in anemia

Blood transfusion therapy is essential support in the treatment of anemia. This therapeutic approach, however, is exercised with a careful balance between the benefits of sustaining oxygen delivery and the intrinsic risks associated with the transfusion of blood. Clinical manifestations of anemia vary with the severity and duration of the condition and many other body compensatory mechanisms, like changes in blood volume and cardiac output [16].

Chronic anemia is often well tolerated by patients given several physiological adaptations. These comprise increased cardiac output due to the expansion of the intravascular plasma volume, vasodilatation, and enhanced flow due to the fall in viscosity. Furthermore, increasing the RBC 2,3-diphosphoglycerate through a right shift of the oxygen dissociation curve allows better oxygen off-loading to peripheral tissues. Nevertheless, some of the persistent symptoms are pallor, tension, dizziness, headaches, vertigo, tinnitus, dyspnea, and decreased activity levels [17].

Chronic, medically related anemia has traditionally been treated with RBC transfusions, but such therapy has been identified as medically overused and sometimes misused by multiple medical societies and accrediting bodies, including the Joint Commission, the American Board of Internal Medicine [18], the American Medical Association, the American Society of Hematology (ASH) and the American Association of Blood Banks. Consequently, several medical societies have taken the initiative to provide guidelines on which to administer RBC transfusion therapy to adult patients [19] and even children [20].

Evolving perspectives on hemoglobin thresholds and transfusion practices

Hemoglobin Levels and Cardiac Output

In adults, the symptomatic threshold for anemia is set at hemoglobin levels that are less than two-thirds of the normal, significantly less than 9-10 g/dL. A threshold limit such as this implies that basal cardiac output increases compensatorily in the setting of lowered blood oxygen-carrying capacity with symptoms that refer to increased cardiac workload like fatigue, dyspnea, and palpitations [21].

Historical Transfusion Methods

Transfusion of red blood cells is the long-standing approach to managing mild to moderate anemia. RBC transfusions were indicated either prophylactically or for symptomatic management of anemic symptoms. Preoperative and perioperative transfusion in a patient with Hb levels below 8-10 g/dL was a standard recommendation. It was presumed that higher Hb levels would diminish perioperative risks and increase survival rates [22].

Change in Transfusion Practice

The outlook on transfusion practices turned around due to concerns about the risks of blood transfusions, particularly the transmission of HIV and other blood-borne infections. Research in cohort populations such as the Jehovah's Witness patient population who refuse blood transfusion on the grounds of religious beliefs reports that morbidity and mortality do not begin to rise substantially until Hb levels fall very low [23], which led to a reassessment of safe transfusion limits.

Critical Hemoglobin Thresholds

Data from Jehovah's Witness patients and other evidence show that critical hemodilution, in which the Hb level is low enough to decrease oxygen consumption due to insufficient oxygen delivery, usually occurs when the Hb level has decreased around 4 g/dL [24]. This has been supported by further studies, including those conducted in Uganda on children with sickle cell anemia or malaria, which showed that the reduction in Hb can be survived and adapted to without detecting any short-term functional implications [25].

Anemia management in cardiovascular disease (CVD)

Increased Perioperative Mortality

Patients with CVD are particularly susceptible to the development of anemia. Indeed, some studies have demonstrated that mortality in the perioperative period is significantly higher for anemic patients with CVD as compared to those without CVD [26]. Thus, by extrapolation, these patients are derived to be managed more specifically for anemia, maintaining a more moderately higher Hb level to ensure adequate delivery of oxygen to the heart and other vital organs [27].

Evidence from Studies

Post hoc analysis and editorials: In a post hoc analysis of a large study, Hébert et al. [28] showed worse outcomes in patients with pre-existing heart disease in the restrictive transfusion strategy group. It is quoted as “survival tended to decrease for patients with pre-existing heart disease in the restrictive transfusion strategy group, suggesting that critically ill patients with heart and vascular disease may benefit from higher Hb [27].”

Clinical practice guidelines: Past clinical practice guidelines have agreed that the critical factor for deciding the tolerance level of a patient toward low Hb levels is the presence of a coronary artery condition. This means that patients with coronary conditions may be considered for transfusion to higher thresholds to minimize the risk of cardiac events. It is quoted as “the presence of coronary artery disease likely constitutes an important factor in determining a patient’s tolerance to low Hb [29].”

Findings of a retrospective study of elderly (>65 years of age) over 79,000 patients admitted for AMI in the United States found that the restrictive blood transfusion strategy was significantly associated with lower mortality rates in this patient group when the admission hematocrit values were lower than 33% [13]. This represents an increased threshold for more active strategies of blood transfusion among the elderly with cardiac disease [30,31].

Evidence From Randomized Controlled Trials, Meta-Analyses and Systematic Reviews

A large Cochrane systematic review of prospective randomized trials [32] compared "high" versus "low" Hb thresholds across 19 trials involving 6264 patients. Key findings were patients without severe cardiac disease adjusted well to the low Hb thresholds. In this group of patients, the transfusion requirements were reduced by 34% (CI 24%-45%). In these trials, there was an average of 1.2 units less RBC transfused (CI 0.5 to 1.8 units) in the low Hb cohorts. A more recent meta-analysis found that a liberal approach to RBC transfusion reduced the incidence of cardiac events, rebleeding, bacterial infections, and mortality [33].

Primary and Major Clinical Trials

TRICC trial: This landmark RCT demonstrated that critically ill patients could tolerate both a restrictive transfusion strategy, targeting a Hb 7-9 g/dL, with statistically fewer RBCs being used, and a more liberal transfusion strategy for a Hb 10-12 g/dL. There was no difference in 30-day mortality between groups, indicating that lower thresholds are safe for the critically ill. While overall 30-day mortality rates were similar between the restrictive and liberal transfusion groups, subgroup analyses revealed potential concerns for patients with significant cardiac disease. Specifically, the results indicated that patients with clinically significant cardiac disease did not benefit from the restrictive strategy, as their mortality rates were not significantly different from those in the liberal strategy group (20.5% vs. 22.9%, P=0.69). This suggests that a more cautious approach may be warranted for this specific subgroup to avoid adverse outcomes [34].

TRACS trial: It was the largest in-cardiac trial, developed for this purpose only, and randomized patients to either restrictive (hematocrit >24%) or liberal RBC transfusion postoperative (>30%). In this study, there was no significant difference in the 30-day all-cause mortality reported by both groups, suggesting that restrictive practice for RBC transfusion is safe within cardiac surgery [35].

FOCUS trial: The Functional Outcomes in Cardiovascular Patients Undergoing Surgical Repair of Hip Fracture (FOCUS) study revolved around the elderly who underwent surgery due to hip fractures whose baseline characteristics include, who had either a history of or risk factors for cardiovascular disease, and whose hemoglobin level was below 10 g per deciliter after hip-fracture surgery. The result of this study is that patients can tolerate an Hb trigger as low as 8 g/dl without an increase in adverse outcomes, thereby supporting a restrictive transfusion practice in this cohort [36].

MINT trial: The Myocardial Infarction and Transfusion trial is a pilot study that was conducted to investigate the liberal transfusion threshold at a hemoglobin level of 10 g/dL, in contrast to a restrictive transfusion threshold at hemoglobin levels of less than 8 g/dL in symptomatic patients with coronary artery disease. Although this set-up was stopped prematurely, preliminary results demonstrated a trend toward higher mortality in the restrictive transfusion cohort, suggesting that these high-risk patients might require higher Hb thresholds. Although the restrictive transfusion strategy was associated with a higher rate of all-cause death compared to the liberal strategy, this difference did not achieve statistical significance, indicating that further investigation may be needed to understand the implications fully. While the MINT trial provides valuable insights into blood transfusion strategies for patients with AMI and anemia, its early termination necessitates careful interpretation of its findings due to issues related to statistical power, potential bias, ethical considerations, and implications for clinical practice [37].

TITRe2 trial: The Transfusion Indication Threshold Reduction (TITRe2) trial was among patients undergoing postoperative coronary artery bypass graft and valve surgery. The study revealed no difference in primary composite ischemic events or infection between restrictive triggers (Hb <7.5 g/dL) and liberal triggers (Hb <9 g/dL). Nevertheless, the restrictive threshold was related to more deaths, suggesting that a higher Hb threshold could be safer for these patients with cardiac issues [38].

REALITY Trial: This indicates that, in patients with AMI and anemia, a restrictive compared with a liberal transfusion strategy resulted in a non-inferior rate of MACE after 30 days. However, the CI included what may be clinically meaningful harm, meaning that the relative risk for the primary outcome was reported as 0.79, with a 1-sided 97.5% CI of 0.00-1.19. This finding satisfies the noninferiority criterion, suggesting that the restrictive strategy is not worse than the liberal strategy in terms of major cardiovascular events [39]. The trials are summarized in Table 1.

**Table 1:** Clinical trials describing results of restrictive vs liberal blood transfusion Table Credits: Ravi Venkata Sai Krishna Medarametla

Reference	Trial	Year	Population	Results	Comments
Hébert et al. [34]	TRICC	1999	Eight hundred and thirty-eight patients of which 418 were assigned to the restrictive strategy and 420 were assigned to the liberal strategy	The 30-day mortality was similar between the groups, but the restrictive transfusion strategy had lower rates among less acutely ill patients and those under 55, with no benefit for those with significant cardiac disease. In-hospital mortality was also lower in the restrictive-strategy group.	The strategy applied in restrictive red-cell transfusion is at least as efficient as, but possibly better than, the liberal strategy of transfusion among critically ill patients, except probably in those with acute myocardial infarction and unstable angina.
Hajjar et al. [35]	TRACS	2010	Five hundred and twelve patients, of which 255 were assigned to the restrictive strategy and 257 were assigned to the liberal strategy	Each additional transfused red blood cell unit increased the risk of complications or death at 30 days	In patients who underwent cardiac surgery, a restrictive perioperative transfusion strategy was non-inferior to the composite outcome of death from any cause or severe morbidity at day 30.
Carson et al. [36]	FOCUS	2011	Two thousand and sixteen patients, of which 1009 were assigned to the restrictive strategy and 1007 were assigned to the liberal strategy	The median number of red cells transfused was two units in the liberal-strategy group and none in the restrictive-strategy group. The rate of the primary outcome was 35.2% versus 34.7%, with similar in-hospital complications and mortality at 60 days.	Compared with a restrictive strategy, a liberal transfusion strategy did not reduce rates of death or inability to walk independently at 60-day follow-up or in-hospital morbidity among elderly patients at high cardiovascular risk.
Carson et al. [37]	MINT	2013	One hundred and ten patients, of which 55 were assigned to the restrictive strategy and 55 were assigned to the liberal strategy	The liberal group had a mean of 1.6 units transfused versus 0.6 in the restrictive group, with the primary outcome occurring in 10.9% versus 25.5% respectively. Death at 30 days was 1.8% in the liberal group versus 13.0% in the restrictive group	Although there was a trend for a more liberal transfusion strategy to involve fewer in-hospital major cardiac events and deaths than a more restrictive strategy, the pilot trial results provide adequate support for the feasibility and need for a definitive trial.
Murphy et al. [38]	TITRe2	2015	2007 patients, of which four withdrew and 1000 were assigned to the restrictive strategy and 1003 were assigned to the liberal strategy	More deaths occurred in the restrictive group (4.2% vs. 2.6%, hazard ratio 1.64, P=0.045), serious complications were 35.7% vs. 34.2%, and total costs were similar.	With respect to morbidity or health care costs, the restrictive transfusion strategy is not better than the liberal transfusion strategy after cardiac surgery.
Ducrocq et al. [39]	REALITY	2021	Six hundred and sixty-eight patients, of which 342 were assigned to the restrictive strategy and 324 were assigned to the liberal strategy	The restrictive transfusion strategy (≤8 g/dL) showed a 30-day composite outcome of 11% versus 14% for a liberal strategy (≤10 g/dL), meeting the noninferiority criterion.	A restrictive transfusion strategy was non-inferior to the liberal strategy for rates of major cardiovascular events in patients who have acute myocardial infarction and anemia. However, the CI did include what may be clinically meaningful harm.

Clinical practice guidelines and implementation

Guidelines on Transfusion Triggers

The recent development in clinical practice guidelines on RBC transfusion reflects an increasing focus on the proper use of blood in healthcare. The selection of a discrete Hb as a "trigger" for RBC transfusion has remained and remains controversial [40]. The guidelines usually recognize that there is a need to consider patient co-variables or other patient-specific criteria for making transfusion decisions [16].

Many published clinical practice guidelines detail the rising concern over appropriate blood utilization. Most commonly, these acknowledge that transfusion is unnecessary at Hb more than 10 g/dL and may be beneficial at less than 6 to 7 g/dL [29,41-48]. This range, therefore, allows a degree of flexibility regarding patient-based factors and clinical judgment.

Professional societies, such as the AABB [49] and the ASH [50], have emphasized that transfusion truly falls under the "Less is More" philosophy [16]. They recommend single-unit RBC transfusions for non-bleeding hospitalized patients and even stress that before additional units are ordered, reassessment of the patient must be done. This concept is designed to avoid the administration of unnecessary transfusions that may carry increased risks [16].

Optimizing red cell utilization

Stanford Health Care (SHC) Initiatives

SHC has made advances in promoting efficient blood utilization with improved physician order entry (POE) using a computerized system and best practice alerts (BPA) to guide blood transfusions. Since the introduction of these measures in July 2010 [51-54], SHC has reduced RBC transfusions by nearly 50% through 2015 without adversely affecting patients [53].

Outcomes such as length of stay, 30-day readmission rates, and mortality have all improved following the use of clinical decision support to restrict transfusion practices [16].

Other Institutional Initiatives

Electronic health records (EHR): EHR systems provide opportunities to improve blood utilization eﬃciently. One center reconﬁgured its POE system to eliminate single-click ordering for two-unit RBC transfusions, making providers select additional units from a droplist. Subsequently, the proportion of 47% of two-unit RBC transfusion was reduced to 15% [55]. One instance is out of 21 medical facilities in Kaiser Permanente Northern California and nearly 400,000 inpatients from 2009 to 2013, the incidents of RBC transfusion were reduced from 14% to 10.8%, and the average pre-transfusion Hb was reduced from 8.1 to 7.6 g/dL, but very importantly, there was no significant variation in these incidents, with the death rate occurring within 30 days over this period [56]. Hence, this states that the restrictive practices of RBC transfusion didn't produce any harmful effect on patients.

Adverse effects of blood transfusion

Blood transfusions are among the most common hospital procedures for hospitalized patients and are associated with significant risks and costs; therefore, healthcare providers must understand the risks associated with blood product administration [57]. While there is growing awareness of the clinical efficacy of restrictive transfusion thresholds in some settings, prompting providers to consider alternatives to transfusion and make treatment decisions to avoid unnecessary transfusions is an essential part of care for specific patient populations [49]. Transfusion reactions represent the most common complication associated with administering blood products and have been known to occur in as many as 1 in 100 transfusions. A transfusion reaction can cause severe discomfort for the patient and an additional cost burden to the healthcare system [58-60]. Although rare, reactions can be fatal, with approximately one in 200000-420000 units transfused associated with death [61]. Given this variety of risks, information respecting the nature, definitions, and management of transfusion-related adverse events should be readily available to clinicians.

There is a tremendous variation in the prevalence of various transfusion reactions per 100,000 units transfused. Allergic transfusion reactions are as high as 112.2 per 100,000 units, while anaphylactic transfusion reactions stand at a low of 8 per 100,000 units. Acute hemolytic transfusion reactions have been reported at 2.5-7.9 per 100,000 units, while delayed hemolytic transfusion reactions occurred at 40 per 100,000 units. The estimated prevalence for delayed serological transfusion reactions is 48.9 to 75.7 per 100,000 units. Compared to this, the febrile nonhemolytic transfusion reaction forms a much more significant chunk, a rate of 1,000 to 3,000 per 100,000 units. Data regarding the prevalence of the hyper hemolytic transfusion reaction is yet unknown. Hypotensive transfusion reactions ranged from 1.8 to 9.0 per 100,000 units. Massive transfusion-associated reactions include those due to citrate, potassium, or cold toxicity; data regarding the prevalence of these is yet unknown. The prevalence rates for post-transfusion purpura and transfusion-associated necrotizing enterocolitis are unknown. The prevalence of septic transfusion reactions ranges from 0.03 to 3.3 per 100,000 units, depending on the product. Transfusion-associated circulatory overload occurs in 10.9 per 100,000 units, and transfusion-related acute lung injury occurs in 0.4 to 1.0 per 100,000 units, with mitigation strategies in place dependent upon the component and post-implementation of the risk mitigation strategies. When methods for irradiation or pathogen reduction are employed, transfusion-associated graft-versus-host disease would be sporadic, near 0% [62]. 

Cost benefits and peer performance review

Tremendous cost savings have been made by implementing restrictive transfusion practices at SHC, which reported a direct reduction of $1.6 million per annum due to improved blood utilization strategies. This figure specifically reflects the cost reduction achieved at SHC from 2012 compared to 2009, highlighting the financial impact of these practices within a single hospital setting [51]. The overall transfusion costs, including laboratory testing, reagent costs, nursing time, and monitoring, have been estimated to be 3.2 to 4.8 times the purchase cost [63]. In conclusion, the savings from reduced transfusion practices overall make a sizable difference. Peer review performance committees are a core foundation for building, maintaining, and achieving excellence in blood utilization performance. These committees analyze providers' performance relative to institutional guidelines and serve as an essential component of continuous education and feedback, the by-product of which is the reduction of variability in transfusion practice and acceptance of only clinically justifiable exceptions [64].

## Conclusions

Issues of efficacy and safety as far as blood transfusion protocols go in MI remain a critical but very contentious issue. Comparing the restrictive versus liberal transfusion strategies reveals that although both methods have their own merits, the choice of strategy has to be tailored to individual patient's conditions. Several RCTs and systematic reviews provide data that demonstrate that a restrictive blood transfusion strategy is at least as effective, or even non-inferior, to various liberal strategies for the reduction of blood transfusions required without compromising patient outcomes in most settings. However, some high-risk groups of patients, mostly with cardiovascular comorbidities, might benefit from more liberal transfusion practices to avoid low hemoglobin-related adverse events.

Coupled with advancements in practice and institutional initiatives, implementing restrictive transfusion guidelines has been shown to decrease costs and reduce variability in transfusion practices. Despite these advantages, though, considering the risks involved with blood transfusion-transfusion reactions and other complications mandates careful consideration and places patient-specific decision-making at the front. It is in the final analysis, while a restrictive approach may be more favored, that AMI and significant cardiovascular disease demand a more individualistic application of transfusion strategies. Continued research and the formation of more and more refined guidelines will have to be undertaken to optimize transfusion practice for efficacy and safety in patients with MI.
